# Complete Genome Sequences of *Legionella pneumophila* subsp. *fraseri* Strains Detroit-1 and Dallas 1E

**DOI:** 10.1128/genomeA.01525-16

**Published:** 2017-02-02

**Authors:** Brian H. Raphael, Natalia A. Kozak-Muiznieks, Shatavia S. Morrison, Jeffrey W. Mercante, Jonas M. Winchell

**Affiliations:** Respiratory Diseases Branch, Division of Bacterial Diseases, National Center for Immunization and Respiratory Diseases, Centers for Disease Control and Prevention, Atlanta, Georgia, USA

## Abstract

We report here the complete genome sequences of two of the earliest known strains of *Legionella pneumophila* subsp. *fraseri*. Detroit-1 is serogroup 1 and was isolated from a lung biopsy specimen in 1977. Dallas 1E is serogroup 5 and was isolated in 1978 from a cooling tower.

## GENOME ANNOUNCEMENT

*Legionella pneumophila* can colonize manmade water systems and, when present in aerosolized water, the organism can be inhaled by susceptible individuals, resulting in a potentially fatal form of pneumonia known as Legionnaires’ disease (1). The species is genetically diverse and contains three known subspecies: *L. pneumophila* subsp. *pneumophila*, *L. pneumophila* subsp. *fraseri*, and *L. pneumophila* subsp. *pascullei* (2). Strains Detroit-1 and Dallas 1E represent *L. pneumophila* subsp. *fraseri*. Detroit-1 was isolated in 1977 from lung biopsy material from a kidney transplant patient who died after developing pneumonia (3, 4). While the strain is serogroup 1, early studies (including DNA-DNA hybridization) revealed that it was genetically distinct from representatives of other serogroup 1 strains (2, 5, 6). Dallas 1E was isolated from a cooling tower sampled during a Legionnaires’ disease outbreak investigation in Dallas, TX in 1978 (7). Dallas 1E is a serogroup 5 type strain and was also recognized as belonging to *L. pneumophila* subsp. *fraseri* (2, 5).

Both strains were sequenced using the Illumina MiSeq (San Diego, CA, USA) and Pacific Biosciences RSII (PacBio; Menlo Park, CA, USA) platforms. The PacBio data were assembled into a single-contig sequence using HGAP 3, and Illumina reads were mapped to this sequence to ensure that nucleotide accuracy was >99.9%. The total genome length was 3,515,377 bp for Detroit-1 and 3,500,943 bp for Dallas 1E. Prokka version 1.8 was used to predict 3,126 (Detroit-1) and 3,101 (Dallas 1E) protein-coding sequences (8). Additionally, both isolates contained 44 predicted tRNAs. The pairwise average nucleotide identity (ANI) (9) between strains Dallas 1E and Detroit-1 was 99.26%. In addition, the pairwise ANI between these two isolates and D-7119 (a representative of *L. pneumophila* subsp. *pascullei*) was ~93.55%, while the pairwise ANI between the same two isolates (Dallas 1E and Detroit-1) and Philadelphia-1 (*L. pneumophila* subsp. *pneumophila*) was ~91.89%. Although 95% ANI has been proposed to delineate bacterial species (9), the *L. pneumophila* subsp. *fraseri* sequences displayed lower ANI with sequences of *L. pneumophila* subsp. *pneumophila* and *L. pneumophila* subsp. *pascullei*, illustrating the high genetic diversity of this species.

These genomes may be useful to researchers examining the molecular basis for serogroup differences between related strains similar to that reported among *L. pneumophila* subsp. *pascullei* isolates (10). Moreover, these complete reference sequences may help support further analysis of *L. pneumophila* subsp. *fraseri* isolates, such as those recovered recently from an outbreak in Portugal (11).

### Accession number(s).

The genome sequences reported here have been deposited at GenBank under GenBank accession numbers CP017457 and CP017458 for Detroit-1 and Dallas 1E, respectively.
